# Availability and type of energy regulate the global distribution of neritic carbonates

**DOI:** 10.1038/s41598-023-47029-4

**Published:** 2023-11-11

**Authors:** Or M. Bialik, Giovanni Coletti, Luca Mariani, Lucrezi Commissario, Fabien Desbiolles, Agostino Niyonkuru Meroni

**Affiliations:** 1 Institute of Geology and Paleontology, University of Münster, Corrensstr. 24, 48149 Münster, Germany; 2 Dr. Moses Strauss Department of Marine Geosciences, The Leon H. Charney School of Marine Sciences, University of Haifa, 31905 Carmel, Israel; 3 Department of Earth and Environmental Sciences, University of Milano-Bicocca, Milan, Italy; 4 CIMA Research Foundation, Savona, Italy

**Keywords:** Biogeography, Palaeontology, Sedimentology

## Abstract

The study of carbonate rocks is primarily reliant on microfacies analysis, which is strongly based on the comparison with modern allochem assemblages. Despite the existence of several models aimed at comprehensively explaining, on the bases of abiotic factors, the distribution of carbonate-producing organisms, a global, quantitative and standardized overview of the composition of shallow-water carbonate sediments is still missing. Aiming to address this gap in knowledge, the current study provides a global database of the available quantitative data on neritic carbonate sediments. This is paired with satellite-based observations for the abiotic parameters. The results highlight a non-linear, multi-variable, dependence in the distribution of allochems and suggest that depth, temperature, and trophic state are, to a certain extent, interchangeable. The implication of which is a level of non-uniqueness for paleoenvironmental interpretation. The resulting distribution is rather continuous and stretches along an energy gradient. A gradient extending from solar energy, with autotrophs and symbiont-bearing organisms to chemical energy with heterotrophs. Further, quantitative data from modern oceans are still required to disentangle the remaining elements of uncertainty.

## Introduction

Shallow-water carbonate-producing settings are some of the most diversity-rich and carbon-absorbing localities on Earth. Yet their fate in light of changes to ocean chemistry and climate is highly debated^1,2^. The Earth’s past is a key window into how these systems will respond to such dramatic changes. However, these environments do not preserve geochemical signals well, making inferences from the constituents and texture—i.e., microfacies—a critical means of investigation^3–6^. A microfacies is the total of all sedimentological and paleontological data that can be inferred from thin sections, peels and polished slabs or rock samples^7^.

The interpretation of these microfacies is, supposedly, based on the comparison with the modern marine environments. Initially, allochem assemblages were classed together into large boxes such as chlorozoan or foramol^8^. As more data became available, further segmentation was required to account for the wide variability observed in nature^9,10^. More recently, various conceptual models, aimed at comprehensively explaining allochem assemblages based on the main abiotic factors, were proposed by several authors^6,11–18^. These models significantly changed our understanding of where and how carbonate sediments are produced, leading to the recognition of a continuum of productive sites along shelves and highlighting an extremely complex framework created by the balance of the main abiotic parameters.

Despite significant progress, there is still a need for a uniform approach for studying modern carbonates. We also still lack a standardized database on global carbonate production that can be used to effectively test models and hypotheses on the effects of the different abiotic parameters. The current approach has been too often to either look at the end members through a broad qualitative view, or to investigate a local dataset and infer from it on a global scale. The former without examining the details; the latter without properly accounting for the existing variability displayed by global carbonate-producing assemblages. Data on modern allochemical assemblages is very rarely reported in a detailed fashion. Various types of allochems are lumped into large categories based on the preferences, biases and focus of the researchers, encompassing groups with largely different ecological requirements. This becomes especially problematic for groups like benthic foraminifera and corals that include both heterotrophic and mixotrophic symbiont-bearing organisms. Descriptions are also widely different among different authors. Crucial elements such as the amount of mud/fines (< 63 μm in size) and the amount of the terrigenous fraction are seldomly reported. Often, only information relative to the facies are provided, while the details of the various sampling stations are not available. This results in an artificial reduction of the internal variability of any examined system. Raw data is also very rarely available, even in recent papers. Meanwhile, there are innate challenges comparing biological and ecological assemblages to sedimentary assemblages due to the high abundance of organic mass in the former. Combined, these problems significantly hinder large-scale analysis and make robust tests of facies models difficult.

Several attempts have been made to glean insights on the future evolution of marine environments from the global record of past shallow-water carbonates^19–23^. Given the short-term scale of the changes we are currently observing in modern environments^24,25^, the record of the past becomes extremely valuable for extrapolating current trends, assessing their causes, and devising long-term reaction strategies^26,27^. However, our limitations in the comprehension of modern systems resulting from the absence of quantitative data greatly hinder these efforts. To address these issues, this study attempts to compile and standardize a comprehensive database—one which encompasses as much available information on modern marine neritic carbonate sediments. Although this database cannot overcome several of the inherent shortcomings of the underlying data, it does provide a broad overview of the currently available information, highlighting the limits and suggesting possible ways for future improvement. Taking into account as much as possible of the total variability of neritic carbonate systems, this work aims to inform on some of the mechanisms that regulate allochem assemblages—without any pre-existing expectations or a priori assumptions.

## Methodology

**Figure 1:**
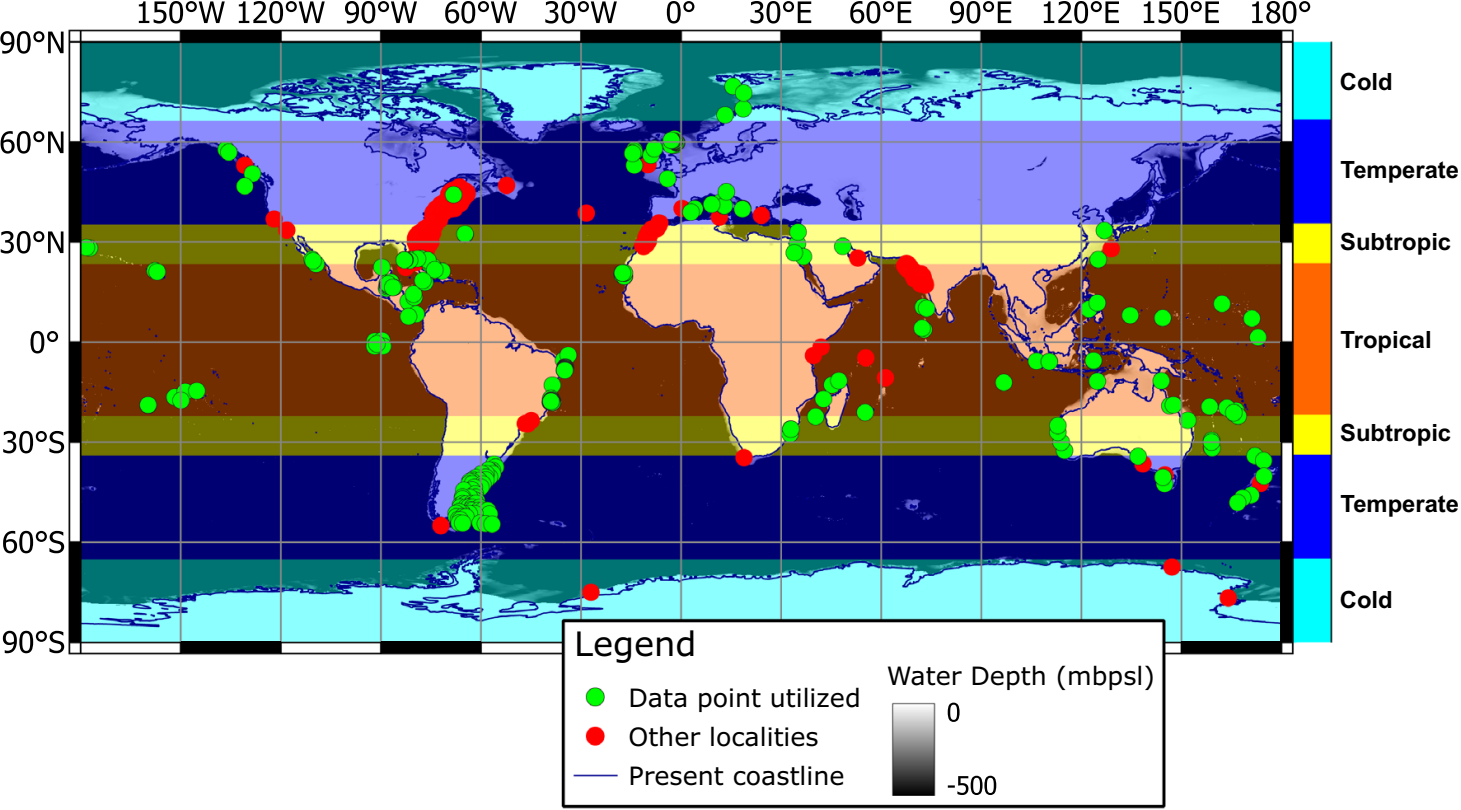
Location map of data points aggregated for this study and delineation of the climate belts discussed in the text. Green points are locations for which sufficient information was available; red points are locations for which some description exists, but no quantitative data. Abbreviation mbpsl stands for meters below present sea level. Map generated with QGIS 3.28.1.

Information on carbonate sediments composition from modern marine environments was aggregated from multiple sources, including data repositories, peer-reviewed papers, books, theses, and reports, totaling 3730 sampling stations (Fig. 1). For 2264 of them, clear quantitative information on the grain composition could be extracted, and 2062 were within the depth bracket of euphotic and mesophotic zones (here set to 200 m). Consistently, the skeletal assemblage of these samples was largely dominated by the benthic component. Of these, 2034 localities were paired with satellite-based time series to assess the time-averaged effect of the abiotic factors. Satellite-based abiotic parameters tested here include: sea surface temperature (SST), chlorophyll α concentrations (Chlα), light attenuation coefficient for a wavelength of 490 nm (KD), and available light at depth (SW). In addition, water depth (WD) and latitude (LAT) were included as abiotic parameters. Multiple sources did not have the information in a tabular form but rather as graphical representation (e.g., pie charts, column charts, map symbols). These and non-digital tables in older sources have been manually digitized and integrated into the database. The different sources had no consistency in the categories reported, with different elements separated, grouped together or completely unreported. Multivariate statistical analysis of allochem categories (see below) and abiotic parameters was then performed. Since the distributions of the abiotic parameters were not normal, the median of the seasonal cycle was used as the centrality index rather than the mean. Analyses were implemented to the dataset using R and PAST softwares^28,29^. Additional spatial analyses were performed with QGIS. Data preparation followed the recommended workflow outlined in Bialik et al.^30^. A detailed explanation of the methods and methodology is provided in Supplement 1.

### Biases, limitations and mitigation

The chosen categories represent the least common denominator among the different sources (Supplement 1). They are devised to minimize zeros in the database, ensuring compatibility between the various reports, all without excessively reducing the underlying complexity of the system. Some uncertainties exist with regards to the fact that zeros in the original source could be either true zeros (absence) or false zeros (category not included), which could bias any statistical analysis of the data^31^. Categories highly skewed by zeros result in several issues with utilizing a database for statistical analysis. The zeros highly bias the averages and may result in false correlation due to overlapping samples with zero values. Additionally, when executing any type of ordinations, highly zero biased samples will lump together, even if in all the other variables, they are dissimilar (clustering due to method). To address this issue various allochem categories were consolidated into larger categories. Reasoning for the selected categories and their constituents is provided in Supplement 1. On these bases, we grouped observative data on the benthic allochem assemblage into 9 consistent categories, which could be established for most data sources (Supplement 1): Molluscs (Mol), symbiont-bearing colonial corals (SBCC), red calcareous algae (RCA), benthic foraminifera (FB), *Halimeda* (Hal), echinoderms (Ech), sessile benthic filter feeders and other sessile heterotrophs (SBFF; including bryozoans, barnacles, serpulids, brachiopods, deep-water corals and sponges), mobile arthropod (MA), bioclasts (BC; other types of bioclasts non identified in the source material) and non-skeletal grains (NSG).

The different reporting approaches between the different sources (Supplement 2) resulted in some elements not always being reported (or in being reported in wildly different fashions). These notably include, among other, grain size, fish detritus, terrigenous fraction, and lime mud abundance. Due to this, these categories were excluded from the statistical analysis. The abundance of lime mud and of terrigenous material are particularly relevant given the influence of terrigenous supply on benthic carbonate producers^32^ and the importance of lime mud as a possible marker of high-temperature/high alkalinity^33,34^ as well as its relationship with microbial^18,35^ and green algal carbonate production^36,37^. Another notable bias is related to benthic foraminifera, which are almost invariably reported as a single category, notwithstanding the large differences that exist between symbiont-bearing and non-symbiont-bearing foraminifera or between porcelaneous and hyaline foraminifera^37^. The different ways the original authors produced and reported the data also introduce biases to the dataset which are hard to account for or may be unknown. An additional bias relates to sample distribution, with only 16% of samples collected north or south of 30° and many areas (e.g., Persian Gulf, Red Sea, South China Sea, Gulf of California) being under-sampled.

To account for all these uncertainties, several ordination analyses were carried out, trying different grouping of the allochems (e.g., separating or lumping sessile heterotrophs), and excluding certain categories (Supplement 1). To provide the reader with a full perspective of the data, both the original information as reported and the processed data used in the analysis are available in Supplement 2.

## Results and discussion

The dataset represents information on allochem assemblages from all continents (excluding Antarctica, Fig. 1) and a wide range of environmental conditions, from very cold to very warm (− 0.2 °C to 29.7 °C of SST), as well as from extremely oligotrophic to mesotrophic. Only 427 samples are from euphotic depth (< 30 m), while most of the samples are from mesophotic conditions. Despite that, the average depth of the samples was 21.2 ± 31 m below present sea level. Light radiation flux (light availability) ranges between 252.7 W/m^2^ and 0.0 W/m^2^ (${\overline{\text{x}}}$ = 99.63 ± 73.4 W/m^2^).

**Figure 2:**
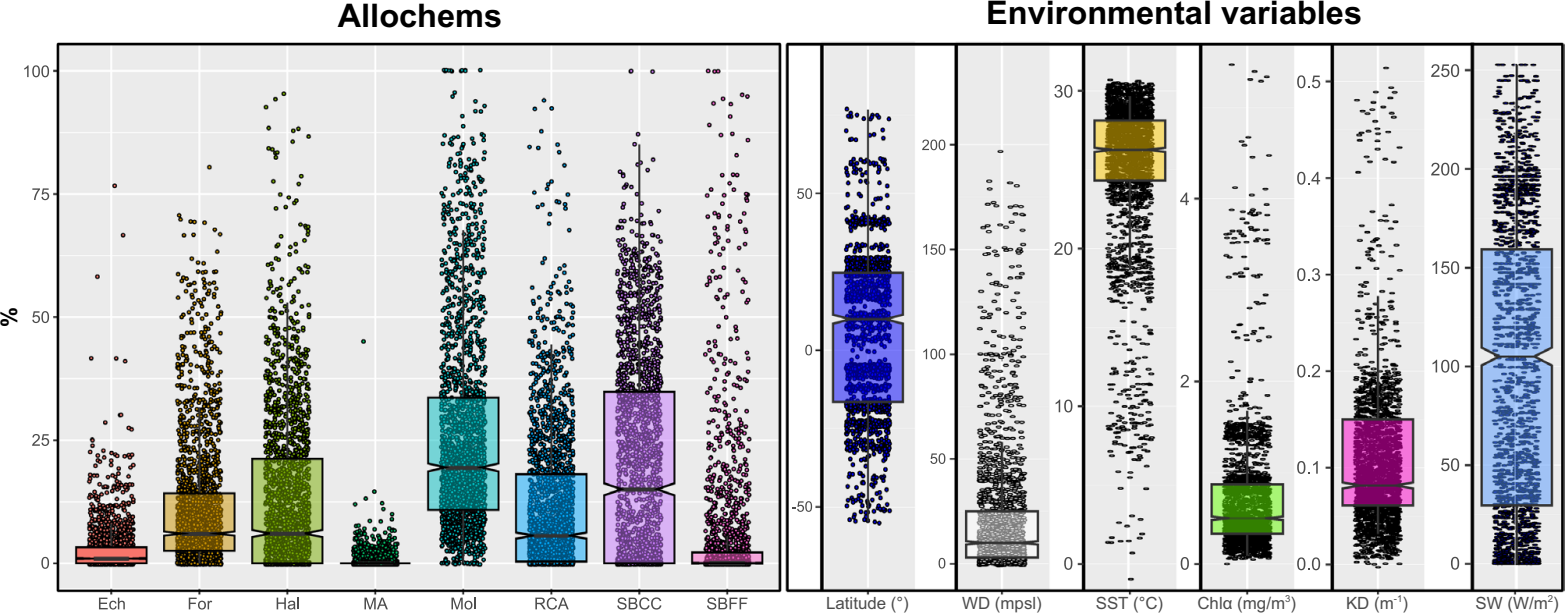
Box and whiskers plot showing the range of all the variables discussed in this study. The center of the box represents the median with the boxes ranging from the 25th to the 75th percentiles. Abbreviation mbpsl stands for meters below present sea level. See text for other abbreviations.

The most abundant allochems in the data set (Fig. 2) are molluscs (${\overline{\text{x}}}$ = 26.6 ± 20.8%), followed by SBCC (${\overline{\text{x}}}$ = 20.2 ± 20.9%). Molluscs occurred in nearly all samples (98% of samples), whereas SBCC were reported only between 29° S and 32° N (74% of samples). The abundance of molluscs is particularly significant. Quantitative estimates of the abundance of carbonate producers in Cenozoic tropical shallow-water settings show that this group is much less abundant even in recent geological records^23^. This emphasizes, as already pointed out by several authors^38–40^, the extent to which dissolution is capable of distorting fossil assemblages and shaping the relatively recent geological record.

The least abundant allochems are MA (${\overline{\text{x}}}$ = 0.5 ± 1.7%) and echinoderms (${\overline{\text{x}}}$ = 2.6 ± 5.0%). Benthic foraminifera and RCA occur in 90% and 74% of samples, respectively, but usually in low abundance (${\overline{\text{x}}}_{{{\text{For}}}}$ = 11.1 ± 13.3%; ${\overline{\text{x}}}_{{{\text{RCA}}}}$ = 11.7 ± 15.0%). Other carbonate producers were reported in the majority of samples, but they represent, on average, less than 2% of the assemblage. NSG were reported only in 32% of the samples. It is unclear if the low occurrence of NSG is due to the terminology used, under-reporting or their actual absence. Here the assumption is made that the NSG are absent in the absence of any additional information.

**Figure 3:**
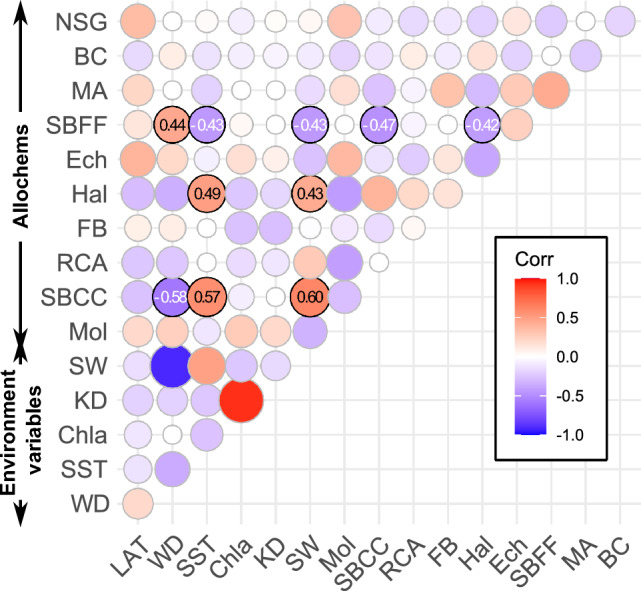
Correlation matrix with Spearman’s ρ for all the variables analysed in this study. Note that SW is a function of WD while KD is a function of Chlα, and as such, their correlation is expected. Red indicates a positive correlation, and Blue negative. Cells with no statistical significance were left uncolored. See text for abbreviations.

Analysis of the correlations between different allochem categories shows little to no correlation between the different categories nor between the categories and abiotic parameters (Fig. 3). Despite that, the size of the dataset allows the detection of statistically significant correlations (p-value < 0.01) even for relatively low correlation coefficients. The most robust correlation observed is between SBCC, water depth (ρ = − 0.58), SST (ρ = 0.57), and light availability at depth (ρ = 0.60). This is well consistent with known limitations on SBCC habitat range^41^. *Halimeda* is the only other group which exhibits a similar relationship with light availability and temperature but with a lower coefficient. In contrast, SBFF exhibits the mirror image: positive correlation to water depth (ρ = 0.44) and negative to SST (ρ = − 0.43) and light availability (ρ = − 0.43).

**Figure 4:**
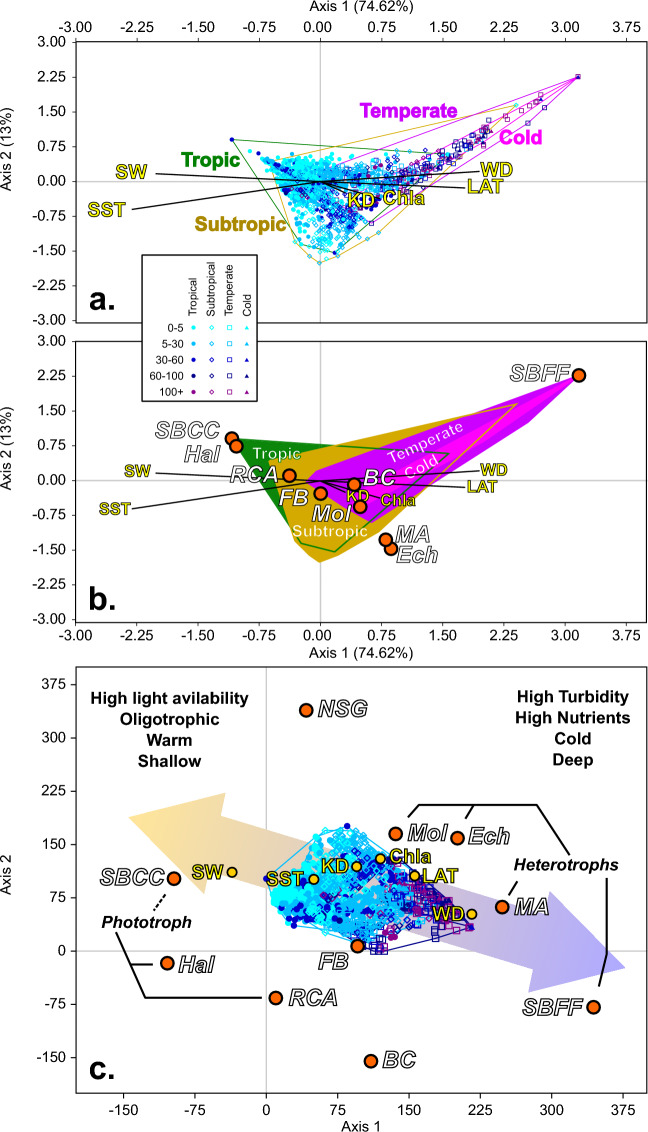
Ordination analyses. (**a**) CCA analysis showing the data points with respect to environmental variables and climate belts grouping. (**b**) As in (**a**) but showing allochems vectors without data points. (**c**) DCA analysis with all variables and data points, specific allochems groups are noted; arrow and additional text are for visualization.

The absence of single-variable correlations here is taken as an indication of non-linear multi-variable dependence in the distribution of allochems. As such, to infer a relationship a multi-variant analysis is required. Multiple ordination methods have been applied to the dataset (including PCA, DCA, NMDS and CCA; see Supplement 1). In none of these methods, a clear differentiation between “T-Type” and “C-Type” carbonate factories^42^ was observed. Rather, all analyses exhibited a continuous gradient. Most of the variance in this multi-variant gradient occurred on two main axes, the principal of which was loaded by SBCC, *Halimeda* and RCA (phototrophs) in one direction and by echinoderms, SBFF, MA and molluscs (heterotrophs) in the other direction (Fig. 4). Benthic foraminifera, bioclasts and NSG do not form major loads on that axis. This may be due to the possible biasing in reporting for bioclasts and NSG. For benthic foraminifera, a different bias exists as most reports did not differentiate between larger benthic foraminifera (LBF) and smaller benthic foraminifera. Would a proper differentiation for benthic foraminifera be available, based on their known distributions^43,44^, LBF would likely group with the phototrophs. The environmental variables exhibit similar variability, with one main axis with SST and light in one direction (associated with the phototrophs), vs. KD, chlorophyll α, latitude (representing climate belts), and water depth in the other (associated with the heterotrophs) (Fig. 4). Water depth and latitude mostly converge, with higher latitude assemblages overlapping with deeper water assemblages. None of the supplementary ordination analyses, using different configurations of the allochem matrix, resulted in different gradients.

These results, paired with prior observations^27,28^ challenges classical hydrodynamic zoning, requiring some revaluation of microfacies interpretation. The observations here suggest that depth, temperature, and trophic state are essentially interchangeable to a certain extent, resulting in a level of non-uniqueness. That is to say, for example, that the loss of a phototrophic group in an assemblage (e.g., SBCC), may be due to cooling or sea-level rise or turbidity. This does not mean that there is no environmental information inferable from the allochem assemblage—rather that multiple factors can cause the same change. Datapoints are aligned along a gradient (Fig. 4) that includes light availability, temperature and chlorophyll (the latter representing available biomass for harvesting)—all the forms of energy. This suggests that energy is a key parameter in determining the composition of the allochem assemblage. Energy availability and utilization dictate physiology^29^. Organisms which can harness solar energy directly prevail in environments where its availability (either directly or in the form of temperature) is the highest. Organisms which obtain their energy from chemical sources (e.g., the breakdown of sugars or other organic molecules sourced from other organisms) prevail where the availability of these is highest and do not compete with the former group in their optimal habitat. Therefore, allochem assemblage can inform directly on the trophic state of the environment, but caution must be applied when extrapolating this to relative sea-level changes if no geometrical, paleogeographical or paleontological information is available.

The relevant influence of the so called “energy resources” (in the sense of metabolic energy, including photosynthetic available energy, chemical energy from the breakdown of organic molecules, and thermal energy from external sources) as well as parameters regulating chemical kinetics (e.g., temperature, salinity, pressure) has been noted in both modern and ancient carbonate depositional systems^16,18,27,45–53^. That said, often only the role of a single energy resource (e.g., light) has been emphasized. As such, the effect of varying the availability of one with respect to the other has not been fully realized. This could be partially explained by the geographic limitations of many of the analyzed studies. Here the synthesis of data from all oceans, latitudes, and light regimes, extending into mesophotic depths, allows a clearer image of the gradients and their relations. Despite that, it is clear that multi-parameter control is in play given the relatively low correlation coefficient at the single variable level (Fig. 3) and the convergence of parameters (Fig. 4). This is clear despite the limitation of the dataset (e.g., lack of data on lime mud, lack of data on the terrigenous fraction, lack of information on the foraminiferal assemblage). The low correlation coefficients may also indicate the underlying effects of other variables that could not be evaluated, such as hydrodynamic energy, salinity, alkalinity, silica, phosphates, nitrates and micro-nutrient abundance. Some of these non-assessed parameters have partial representation in the available abiotic parameters, such an Chalα and nutrients^54^ or hydrodynamic energy and turbidity^55^, expressed in our data set as extinction coefficient. Hydrodynamic energy in particular effects biological processes by influencing food availability, water transparency, and feeding behaviors, as well as taphonomic processes controlling reworking transport and fragmentation of the allochems^56,57^. However, these non-assessed abiotic factors are strongly influenced by local factors that are hard to evaluate using satellites or other remote sensing which are limited in their capacity to observe such aspects. Although some information could be derived from numerical models, these outputs may not be entirely consistent with the satellite record and could introduce additional biases. Nonetheless, it is worth noting that the distribution of the sampling stations within the space of the ordination analysis is only based on the similarities and differences of the allochem assemblages themselves. Regardless of the investigated abiotic parameters, the results shown in Fig. 4 (and additional runs in Supplement 1) represent the global distribution of allochem assemblages based on the currently available quantitative data. This distribution can be explained in terms of “energy resources”. Most samples are distributed along a main axis that clearly strongly reflects “energy resources” availability through indicators such as feeding behavior of the different groups. This is not to say that factors such as inorganic carbon chemistry or hydrodynamic energy do not play a part in generating allochem gradients. But the preference of groups distribution between autotrophs and heterotrophs along the principle axis and the convergence of depth and latitude strongly suggest “energy resources” as the main contributors to the gradient along the main axis.

In the gradient observed in the data, no differentiation could be observed between the “T-Type” and “C-Type” carbonate factories. However, in this context, it is important to highlight that the reporting here refers to allochem assemblages—not to geometries. That said, extrapolating from the findings here and in light of detailed work done on geometries of carbonate deposits^30,31^, the “T-Type” and “C-Type” geometries may similarly represent end-members along a gradient. A gradient that goes from prevailing solar energy to prevailing chemical energy.

These results are still inextricably entangled to the many biases caused by the choice of the categories and the lack of information on important elements, such as the abundance of mud and terrigenous fraction, and taphonomic (notably biostratinomic) processes. These biases cannot be currently resolved given the heterogeneous nature of the original reports. Taphonomy in particular is a problem that is hard to address, due to the loss of shells within the top few cm of the sediment^58^ and on the seafloor^59,60^, which are not well quantified across different settings. Nonetheless, the outcome of the analysis carried out here highlights the massive potential for improving our understanding of the distribution of modern carbonates. Given sufficient information and sufficient high quality data, cause-and-effect explanations could be derived for the characteristics of most carbonate systems^6^.

## Conclusions

A microfacies is the total of all sedimentological and paleontological data that can be inferred from thin sections, peels and polished slabs or rock samples. It is a key tool in paleoenvironmental reconstruction. The results presented here suggest that allochem assemblages exist on a multi-dimensional continuum that does not differentiate between “carbonate factories”. Similarly, there is a convergence between environmental variables such as depth and climate belts. These findings stress the need for more caution in water-depth reconstruction purely based on quantitative microfacies analysis, as allochem assemblages appear to be most dependent on energy availability (either from solar or chemical/biological sources). Qualitative elements, such as sedimentary texture, can help mitigate some of the non-uniqueness in interpretation and should always be considered where possible. Early diagenetic elements and geochemical datasets may similarly contribute.

This work is the first step towards a revaluation of the microfacies paradigm relying on an evidence-based approach. This is still an incomplete endeavor as there is, currently, limited quantitative standardized information for parameters like terrigenous supply, carbonate mud abundance, as well as deconvolution of the distribution of the various types of non-skeletal grains and of benthic foraminifera. This work has also limited itself only to direct measurements. It is likely that more dimensions (such as hydrodynamic energy, alkalinity and nutrients) from models and extrapolations could add further information and further levels of complexity.

We thus implore researchers, both those working on modern and ancient deposits, to embrace a more quantitative and standardized approach and to make their data available. Generally, more data and work on modern and ancient environments, using a quantitative and consistent approach, are required to improve our ability to reconstruct the past, understand the present, and forecast the future.

### Supplementary Information

Supplementary Information 1.

Supplementary Information 2.

## Data Availability

All data will be made available online via repository upon acceptance. All data and additional information on the methods are available online through the FigShare repository at https://figshare.com/s/9e41576427eb4d86350a, and can be cited directly by doi: 10.6084/m9.figshare.23537934.
