# Supplementary material for: Availability and type of energy regulate the global distribution of neritic carbonates
Source: Sci Rep. 2023 Nov 11;13:19687. doi: 10.1038/s41598-023-47029-4 (PMC10640608; doi:10.1038/s41598-023-47029-4)
Supplement: Supplementary file 1 — Supplementary Information 1. [file 41598_2023_47029_MOESM1_ESM.docx]

# Supplement 1

**1.1 Data Collection and Treatment**

Information on carbonate sediments composition from modern marine environments was aggregated from multiple sources, including data repositories, peer-reviewed papers, books, theses and reports. Most of the sources were retrieved online, initially searching for different localities associated with key words (e.g., South Africa + carbonate sediments). Then, the references included in the initially identified group of sources were investigated. Finally, various online tools for paper analysis (e.g., Google Scholar related papers feature; Connected Papers, Research Rabbit) were employed to identify further data sources. Other sources not available online were also taken into account to the best of the author's capabilities. It is safe to assume that further data might exist, especially in the latter group.

This literature review yielded 3730 data-points with varying degrees of quantitative information on the composition of carbonate sediments. In a significant portion of these data-points the information was provided as facies contours, and usually, for each facies, only the abundance of the dominant components, expressed as min-max range, was available. In other instances, the sources included information on the composition of allochem assemblages separated on the basis of grain size, but provided no data on the relative weight of each grain-fraction over the total of the examined sample. These types of sources were excluded from the analysis.

For 2264 data-points, clear quantitative information on the composition of allochem assemblages was available. Wherever possible, for these data-points, accurate coordinates and water depth of the sampling site were retrieved from the original source. Otherwise, coordinates and water depth were inferred from QGIS, by uploading and georeferencing the maps of the sampling sites included in the original source. This method is not perfectly accurate. To account for this problem, we used a relatively large-sized box for retrieving satellite-based data on abiotic parameters (see 1.2). Since the focus of the study are neritic allochem assemblages, only samples from the photic zone (here set at 200 m to accunt for maximum deapth in highly oligotrophic regions) were included, thus further reducing the data pool to 2062 data points (Supplement 2). For 28 of them, satellite data was not fully available, reducing the total of actually used data points to 2034.

The reported categories were inconsistent across the many sources reviewed. Certain elements were separated in some sources but grouped together in others. Certain categories were also frequently not reported. To standardize the dataset and minimize zeros for the multivariate statistical analysis, allochems reported in the various sources were consolidated into 9 consistent allochem groups (Table S1).

**Table S1:** Allochems included in each consolidated category.

| **Allochem groups** | **Included allochems** |
| --- | --- |
| Mollusks (Mol) | Bivalve, Gastropods, Scaphopods, Mollusks |
| Symbiont-bearing colonial corals (SBCC) | Symbiont-bearing colonial corals |
| Red calcareous algae (RCA) | Red calcareous algae |
| Benthic foraminifera (BF) | Large benthic foraminifera, Small benthic foraminifera, Encrusting foraminifera |
| *Halimeda* (Hal) | *Halimeda* |
| Echinodermata (Ech) | Echinoderms, Crinoids, Ophiuroids |
| Sessile benthic filter feeders (SBFF) | Bryozoans, Barnacles, Serpulids, Brachiopods, Deep-water corals |
| Mobile Arthropoda (MA) | Decapods, Arthropods, Ostracods |
| Bioclasts (BC) | Other biogenic allochems, occurring in lesser abundance and whose accurate origin was not indicated in the original source; non identified bioclasts |
| Non-Skeletal Grains (NSG) | Ooids, Peloids, Pellets, Faecal Pellets, Grapestones, Aggregates |

Although this operation introduces a level of abstratization to the database, and possibly some bias due to the apriority selection of the categories, it is a critical step for the statistical analysis. Due to the heterogeneous nature of the original data, both true and false zeros ^1^ are introduced into the data sets. These could lead to unwanted effect such as clustering of data based on methodology rather than a real gradient. For example barnacles (which are reported from most high-latitude site and in a handful of tropical ones), might be reported by paper 1 and while the category would be absent from paper2 (e.g., most of the paper from tropical latitudes). Therefore, in the cell of the matrix relative to barnacles abundance for paper2, we will have a zero. The statistical analysis cannot separate the zero caused by the absence of the category (false zero), from the zero caused by the absence of the allochem (true zero).

Furthermore, categories highly skewed by zeros result in several issues. The zeros highly bias the averages and may result in false correlation due to the overlapping of samples with zero values. Additionally, when executing any type of ordinations, highly zero biased samples will lump together, even if in all the other variables, they are dissimilar (methodology introduced bias). The wildly heterogeneous nature of the source material creates a large number of these zeros, sometimes referred to as “Zero inflation”. For example, for the groups included in the “sessile benthic filter feeders and other sessile heterotroph” category, the original data for the individual categories had 90% of the cells being zeros, this has been reduced to 60% following the consolidaton.

Although the design of the categories represent a bias as it influence the final result of the analysis, it must be stressed that is a general limitation of every scientific analysis as the choice of the reference frame is always bound to influence the observations.

To provide the reader with all the elements useful for evaluating the possible effects of our choices, a discussion on the design of the various categories is here presented. Supplementary material 2 also include the raw data digitized from the source material before any statistical treatment was performed.

In the following are the justification to the selection of the consolidated categories (see Table S1 for abriviations):

**Mol**: Molluscs are one of the most commonly reported type of allochem, occurring the vast majority of data points. However, in the source material, benthic molluscs were often reported lumped together, more rarely separated into gastropods and bivalves, and even more rarely into gastropods, bivalves, and/or scaphopods. Pteropods are rarely reported. When reported together, no information on the relative abundance of the various groups of molluscs was indicated.

**SBCC**: Generally all types of corals were generally reported together. however, given the massive difference among the ecological significance of symbiont bearing and non symbiont bearing corals, their separation was of the utmost importance. In order to be able to understand which type of coral was included into the “coral” category, the source material was investigated for information on the types of observed corals, water depth and type of setting. The performed subdivision between symbiont-bearing colonial corals and the other types of corals is thus related to the source material, and its accuracy reflects the precision provided by the original material. It must be stressed that this is a complex division, as a small coral fragment, resulting from the fragmentation of a colony of a deep-water colonial coral, is morphologically similar to the fragment of a colony of a symbiont bearing colonial coral. This separation is even more complex when using thin sections as the distinctive morphological characters of the type of coral are usually not recognizable.

**RCA**: Red calcareous algae are generally reported with no quantitative indication of the growth form or the type of algae.

**BF**: Benthic foraminifera are almost invariably reported as a single category, often just as “foraminifera”, notwithstanding the large differences that exist between symbiont-bearing and non-symbiont-bearing foraminifera or between porcelaneous and hyaline foraminifera ^2^. Differently from corals, indications on the types of benthic foraminifera are less commonly reported. Furthermore, all localities including symbiont-bearing large benthic foraminifera also display relevant amounts of heterotroph small benthic foraminifera. Therefore, unless significant assumptions are made (e.g., in the images of the sediment presented into the source material, 60% of the foraminifera is represented by symbiont-bearing benthic foraminifera; therefore, 60% of all the reported foraminifera are symbiont-bearing foraminifera), separating the various types of foraminifera, grouped together in the original paper, is impossible.

**Hal**: The only type of green calcareous alga reported as a relevant component of the allochem assemblage is *Halimeda*, with no reporting of other types of green algae was found in most sources. Although other green calcareous algae are known to be important carbonate producers, their skeletal components are too small and fragile and, consequently, their contribution to carbonate sedimentation is mostly in the mud fraction ^3^. The latter is also produced by other organisms and by different taphonomic processes, making differentiation hard if not impossible. This was further complicated by the fact that as nearly all reviewed works did not include a breakdown of constituents in the fine fraction.

**Ech**: Excluding some crinoids, all echinoderms are mobile heterotrophs. Their abundance is reported in the vast majority of the analyzed papers, but usually as a lumped category, with no indication useful for separating them into functional groups. Only a handful of cases reported separately on echinoids, ophiouroids and crinoids. Therefore, all types of echinoderms were lumped together in the analysis.

**SBFF:** The various types of carbonate producers, included in the sessile heterotroph group where combined into a single category (e.g., deep-water corals, non-symbiont bearing corals, brachiopods, serpulids, sponges) as, individually, they are rarely reported. This results in a large number of zeros, likely false zeros, which is a source of difficulty for any statistical analysis ^1^. Due to their common ecological preferences (high-nutrients, sustained hydrodynamic energy), and their sessile lifestyle, they were lumped together. Several runs with different type of grouping for sessile heterotrophs were also attempted (see also below).

**MA**: As with SBFF, mobile arthropods (mainly consisting of decapods, crabs and to a lesser extent, ostracods) were also considered a single category.

**BC**: Most of the reports included other two categories “other” and “non-identified”. The “other” category usually gathers types of carbonate producers that are rare in the study area, and no information is usually provided on the relative abundance of the various types of allochems that occur under this banner. The “non-identified” category lumps together all bioclasts lacking elements useful for the identification, generally with no indications on the causes of the missed identification. Not every paper includes both categories, and often only one is present. In the absence of more detailed information, they have been grouped together as “bioclasts”. The abundance of non-identified bioclasts can be related to taphonomic processes (micritization, fragmentation, abrasion), which in turn can be connected to hydrodynamic energy. However other factors can also be relevant, like poor identification. In order to try to evaluate the effects of some of these problems, attempts at analysing the dataset using only the identified bioclasts (i.e., the “other” category and excluding the “non-identified” category) were also performed.

**NSG**: Non-skeletal grains are usually only reported from tropical latitudes and are often divided in an inconsistent array of sub-categories.

The heterogeneous nature of the original source material is also the cause of the exclusion of elements like the abundance of mud and terrigenous material from the statistical analysis. In the case of the abundance of mud, the latter, when indicated, is reported a wide variety of ways: grain-size analysis not necessarily performed in the same sample for which the composition of the allochem assemblage is provided; percentage of the material whose average grain-size is less than 125 µm; percentage of the material whose average grain-size is less than 63 µm; percentage of “fine debris” with no indication of the threshold for defining a grain as “fine”.

**1.2 Satellite Data**

SST data come from the Level 4 (L4) v2.1 gap-free analysis product of the ESA-CCI (European Space Agency - Climate Change Initiative) project ^4^ (available at https://data.ceda.ac.uk/neodc/esacci/sst/data/CDR_v2/Analysis/L4/v2.1). They are given on a regular grid with a 0.05° spacing and a daily frequency. Monthly mean maps are obtained with CDO (Climate Data Operator). Monthly maps of Chla and KD are retrieved from the ESA-CCI Ocean Colour dataset at 4 km grid spacing ^5^ (available at https://dap.ceda.ac.uk/neodc/esacci/ocean_colour/data/v5.0-release/geographic/netcdf/chlor_a/monthly/v5.0/ and https://dap.ceda.ac.uk/neodc/esacci/ocean_colour/data/v5.0-release/geographic/netcdf/kd/monthly/v5.0/, respectively). Surface shortwave radiation fluxes are retrieved from the ESA-CCI Cloud dataset as monthly maps at 0.5° grid spacing on a regular grid (available at https://public.satproj.klima.dwd.de/data/ESA_Cloud_CCI/CLD_PRODUCTS/v3.0/L3C/AVHRR-AM/AVHRR_METOPA/).

All data cover the time period between January 2008 and December 2013, which is the overlapping period of the satellite records selected. For each sample, a box of 0.2°x0.2° centred on the sample position has been defined. The mean value within such a box of the quantities of interest (except for the shortwave fluxes) has been computed excluding the missing values. In case all values were missing within the 0.2° box, a box of 2°x2° has been used, which is also the size of the box used for the shortwave fluxes. In this way, a monthly time series is available for each sample.
The time series of the available shortwave energy flux at the depth of the sample has been computed with a simple exponential absorption profile, attenuated with the KD coefficient (assumed to be representative for the whole shortwave spectral range), namely:

SW = SW0*exp(-KD*WD)

with SW0 denoting the surface net shortwave energy flux (computed as the difference between the downwelling and the upwelling fluxes). From the full timeseries, a seasonal cycle has been computed for each sample.

The code is available at https://github.com/agonmer/sat4sediments.

**1.3 Statistical treatment**

Statistical analysis was carried out with a combination of R and PAST softwares ^6,7^ (for ordination see https://doi.org/10.6084/m9.figshare.c.5250993.v1). We present only the ordination generated in PAST to maximise reproducibility for those not familiar with coding. A correlation matrix using the Spearman *r* coefficient was generated to detect monotonic relationships among couples of variables in the dataset (Tables S2 and S3). Following correlation analysis, spatial patterns were examined and verified visually in QGIS ^8^.

**Table S2**: Correlation matrix for all variables in this study.

| **Spearman’s ρ** | **LAT (y)** | **Water depth** | **SST** | **Chla** | **KD** | **SW** | **Mol** | **SBCC** | **RCA** | **For** | **Hal** | **Ech** | **SBFF** | **MA** | **BC** | **NSG** |
| --- | --- | --- | --- | --- | --- | --- | --- | --- | --- | --- | --- | --- | --- | --- | --- | --- |
| **LAT (y)** | 1.00 | 0.19 | -0.12 | -0.10 | -0.19 | -0.14 | 0.19 | -0.26 | -0.24 | 0.08 | -0.28 | 0.38 | 0.13 | 0.21 | ### | 0.34 |
| **Water depth** | 0.19 | 1.00 | -0.36 | -0.11 | -0.19 | -0.91 | 0.24 | -0.58 | -0.23 | 0.09 | -0.34 | 0.20 | 0.44 | 0.18 | 0.09 | 0.00 |
| **SST** | -0.12 | -0.36 | 1.00 | -0.25 | -0.23 | 0.48 | -0.10 | 0.57 | -0.08 | 0.19 | 0.49 | -0.06 | -0.43 | -0.19 | ### | 0.02 |
| **Chla** | -0.10 | -0.11 | -0.25 | 1.00 | 0.93 | -0.24 | 0.26 | -0.07 | -0.15 | -0.26 | -0.24 | 0.16 | 0.03 | -0.07 | ### | -0.07 |
| **KD** | -0.19 | -0.19 | -0.23 | 0.93 | 1.00 | -0.16 | 0.19 | 0.01 | -0.10 | -0.27 | -0.17 | 0.07 | -0.03 | -0.03 | ### | 0.01 |
| **SW** | -0.14 | -0.91 | 0.48 | -0.24 | -0.16 | 1.00 | -0.32 | 0.60 | 0.27 | -0.01 | 0.43 | -0.26 | -0.43 | -0.15 | ### | 0.03 |
| **Mol** | 0.19 | 0.24 | -0.10 | 0.26 | 0.19 | -0.32 | 1.00 | -0.27 | -0.41 | -0.09 | -0.42 | 0.36 | 0.08 | 0.16 | ### | 0.31 |
| **SBCC** | -0.26 | -0.58 | 0.57 | -0.07 | 0.01 | 0.60 | -0.27 | 1.00 | 0.23 | -0.15 | 0.38 | -0.12 | -0.47 | -0.25 | ### | -0.08 |
| **RCA** | -0.24 | -0.23 | -0.08 | -0.15 | -0.10 | 0.27 | -0.41 | 0.23 | 1.00 | 0.03 | 0.20 | -0.21 | -0.05 | -0.05 | 0.09 | -0.16 |
| **FB** | 0.08 | 0.09 | 0.19 | -0.26 | -0.27 | -0.01 | -0.09 | -0.15 | 0.03 | 1.00 | 0.14 | 0.13 | 0.13 | 0.32 | ### | -0.10 |
| **Hal** | -0.28 | -0.34 | 0.49 | -0.24 | -0.17 | 0.43 | -0.42 | 0.38 | 0.20 | 0.14 | 1.00 | -0.38 | -0.42 | -0.29 | 0.15 | -0.19 |
| **Ech** | 0.38 | 0.20 | -0.06 | 0.16 | 0.07 | -0.26 | 0.36 | -0.12 | -0.21 | 0.13 | -0.38 | 1.00 | 0.24 | 0.27 | ### | 0.12 |
| **SBFF** | 0.13 | 0.44 | -0.43 | 0.03 | -0.03 | -0.43 | 0.08 | -0.47 | -0.05 | 0.13 | -0.42 | 0.24 | 1.00 | 0.43 | 0.01 | -0.22 |
| **MA** | 0.21 | 0.18 | -0.19 | -0.07 | -0.03 | -0.15 | 0.16 | -0.25 | -0.05 | 0.32 | -0.29 | 0.27 | 0.43 | 1.00 | ### | 0.07 |
| **BC** | -0.16 | 0.09 | -0.12 | -0.07 | -0.05 | -0.08 | -0.18 | -0.11 | 0.09 | -0.08 | 0.15 | -0.19 | 0.01 | -0.22 | 1.00 | -0.18 |
| **NSG** | 0.34 | 0.00 | 0.02 | -0.07 | 0.01 | 0.03 | 0.31 | -0.08 | -0.16 | -0.10 | -0.19 | 0.12 | -0.22 | 0.07 | ### | 1.00 |

**Table S3**: corresponding p value matrix for table S2

| **p value** | **LAT (y)** | **Water depth** | **SST** | **Chla** | **KD** | **SW** | **Mol** | **SBCC** | **RCA** | **For** | **Hal** | **Ech** | **SBFF** | **MA** | **BC** | **NSG** |
| --- | --- | --- | --- | --- | --- | --- | --- | --- | --- | --- | --- | --- | --- | --- | --- | --- |
| **LAT (y)** | 0.00 | 0.03 | 0.00 | 0.01 | 0.00 | 0.00 | 0.00 | 0.00 | 0.00 | 0.00 | 0.00 | 0.00 | 0.00 | 0.00 | 0.00 | 0.00 |
| **Water depth** | 0.03 | 0.00 | 0.00 | 0.77 | 0.00 | 0.00 | 0.00 | 0.00 | 0.00 | 0.00 | 0.00 | 0.00 | 0.00 | 0.09 | 0.00 | 0.00 |
| **SST** | 0.00 | 0.00 | 0.00 | 0.00 | 0.00 | 0.00 | 0.00 | 0.00 | 0.70 | 0.07 | 0.00 | 0.00 | 0.00 | 0.00 | 0.00 | 0.00 |
| **Chla** | 0.01 | 0.77 | 0.00 | 0.00 | 0.00 | 0.00 | 0.00 | 0.00 | 0.00 | 0.00 | 0.00 | 0.00 | 0.00 | 0.51 | 0.00 | 0.03 |
| **KD** | 0.00 | 0.00 | 0.00 | 0.00 | 0.00 | 0.00 | 0.00 | 0.18 | 0.05 | 0.00 | 0.00 | 0.00 | 0.08 | 0.20 | 0.00 | 0.02 |
| **SW** | 0.00 | 0.00 | 0.00 | 0.00 | 0.00 | 0.00 | 0.00 | 0.00 | 0.00 | 0.00 | 0.00 | 0.00 | 0.00 | 0.00 | 0.00 | 0.04 |
| **Mol** | 0.00 | 0.00 | 0.00 | 0.00 | 0.00 | 0.00 | 0.00 | 0.00 | 0.00 | 0.00 | 0.00 | 0.00 | 0.48 | 0.00 | 0.00 | 0.00 |
| **SBCC** | 0.00 | 0.00 | 0.00 | 0.00 | 0.18 | 0.00 | 0.00 | 0.00 | 0.14 | 0.00 | 0.00 | 0.00 | 0.00 | 0.00 | 0.00 | 0.00 |
| **RCA** | 0.00 | 0.00 | 0.70 | 0.00 | 0.05 | 0.00 | 0.00 | 0.14 | 0.00 | 0.00 | 0.00 | 0.00 | 0.00 | 0.01 | 0.01 | 0.00 |
| **FB** | 0.00 | 0.00 | 0.07 | 0.00 | 0.00 | 0.00 | 0.00 | 0.00 | 0.00 | 0.00 | 0.05 | 0.00 | 0.59 | 0.00 | 0.00 | 0.00 |
| **Hal** | 0.00 | 0.00 | 0.00 | 0.00 | 0.00 | 0.00 | 0.00 | 0.00 | 0.00 | 0.05 | 0.00 | 0.00 | 0.00 | 0.00 | 0.00 | 0.00 |
| **Ech** | 0.00 | 0.00 | 0.00 | 0.00 | 0.00 | 0.00 | 0.00 | 0.00 | 0.00 | 0.00 | 0.00 | 0.00 | 0.00 | 0.00 | 0.00 | 0.00 |
| **SBFF** | 0.00 | 0.00 | 0.00 | 0.00 | 0.08 | 0.00 | 0.48 | 0.00 | 0.00 | 0.59 | 0.00 | 0.00 | 0.00 | 0.01 | 0.77 | 0.00 |
| **MA** | 0.00 | 0.09 | 0.00 | 0.51 | 0.20 | 0.00 | 0.00 | 0.00 | 0.01 | 0.00 | 0.00 | 0.00 | 0.01 | 0.00 | 0.01 | 0.09 |
| **BC** | 0.00 | 0.00 | 0.00 | 0.00 | 0.00 | 0.00 | 0.00 | 0.00 | 0.01 | 0.00 | 0.00 | 0.00 | 0.77 | 0.01 | 0.00 | 0.00 |
| **NSG** | 0.00 | 0.00 | 0.00 | 0.03 | 0.02 | 0.04 | 0.00 | 0.00 | 0.00 | 0.00 | 0.00 | 0.00 | 0.00 | 0.09 | 0.00 | 0.00 |

Subsequently, the data set was examined and evaluated, every variable was tested for range and normality. Based on this, all the variables were transformed and standardised (Table S4) prior to ordination. Following both operations all variables tested as normally distributed with p<0.01.

**Table S4**: Transformation and standardisation of all variables in this study prior to ordination analysis.

| **Variable** | **Transformation** | **Standardisation** |
| --- | --- | --- |
| Latitude (°) | \|x\|/100 | ^2^√arcsin(x) |
| Water depth (mbpsl) | x/1000 | ^2^√arcsin(x) |
| SST (°C, Median) | x/100 | ^2^√arcsin(x) |
| Chla (mg*m^-3^, Median) | x/10 | ^2^√arcsin(x) |
| K_D_ (m^-1^, Median) | x | ^2^√arcsin(x) |
| SW (W*m^-2^, Median) | x/1000 | ^2^√arcsin(x) |
| Molluscs | x/100 | ^2^√arcsin(x) |
| Symbiont bearing colonial corals | x/100 | ^2^√arcsin(x) |
| RCA | x/100 | ^2^√arcsin(x) |
| Foraminifera | x/100 | ^2^√arcsin(x) |
| Halimeda | x/100 | ^2^√arcsin(x) |
| Echinoderms | x/100 | ^2^√arcsin(x) |
| Sessile benthic filter feeders | x/100 | ^2^√arcsin(x) |
| Mobile Arthropoda | x/100 | ^2^√arcsin(x) |
| Bioclasts | x/100 | ^2^√arcsin(x) |
| Non-skeletal grains | x/100 | ^2^√arcsin(x) |

Four ordination analyses have been used in this study: Principal Component Analysis (PCA^9^), Nonmetric MultiDimensional Scaling (NMDS; Shepard, 1962), Detrended Correspondence Analysis (DCA^11^) and Canonical Correspondence Analysis (CCA^12^). NMDS (Figure S1) was performed on boths transformation and standardisation as well as non-transformation and non-standardisation using the Manhattan and Gower indexes. In all runs NMDS resulted in very high stress (~1.6). This may be due to the size of the data set, as it is significantly above all reasonable thresholds. As no group separation was found to allow validation of the ordination with a statistical test, the NMDS outputs were not used in the interpretation but were compared to other outputs. PCA (Figure S2) was ran with the abiotic environmental variables present and absent, yielding similar results with PC1 being the dominant component (32.4% variance with environmental variables; 32.7% variance without environmental variables), combination of PC1 and PC2 did not exceed 50% of variance (48% with environmental variables, 49.6% without) however PC3 was below the broken stick. In CCA analysis, a similar dominance of the main axis (74.6%) was also observed.

To account for any issue which may arise from the lumping, multiple different runs of ordination with different configurations have been attempted. Notably, exclusion of foraminifera (Figure S3), of non-identified bioclasts (Figure S4) and alternative lumping of the different types of sessile heterotrophs (Figure S5). All runs resulted in similar configuration: deep and cold settings coalescing at one end of a gradient, shallow and warm settings on the other. Given the ordination of the datapoints, this gradient is likely to be mainly controlled by energy resources availability.


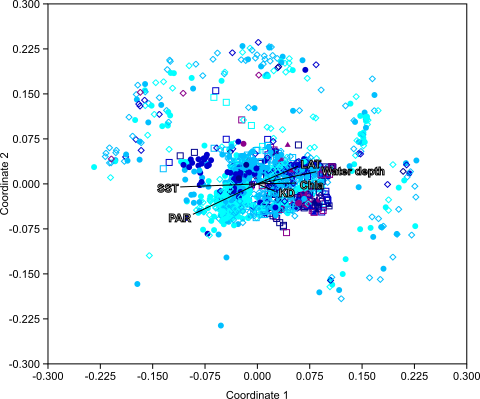


**Figure S1**: Output of NMDS run with non-transformed and non-standardised data using the Manhattan index. See Figure 4 in the main text for legend.


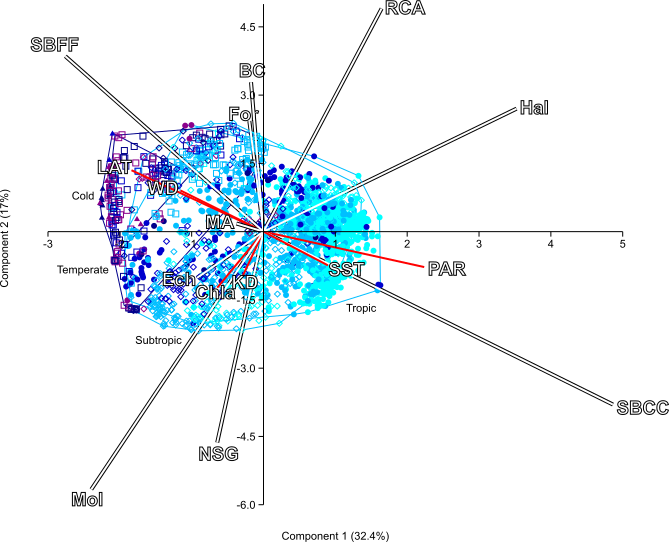


**Figure S2**: Output of PCA run with transformed and standardised data using all variables, presented in eigenvalue scale. Environmental variables are noted in red and allochems in black and white for visualisation only. See Figure 4 in the main text for legend.


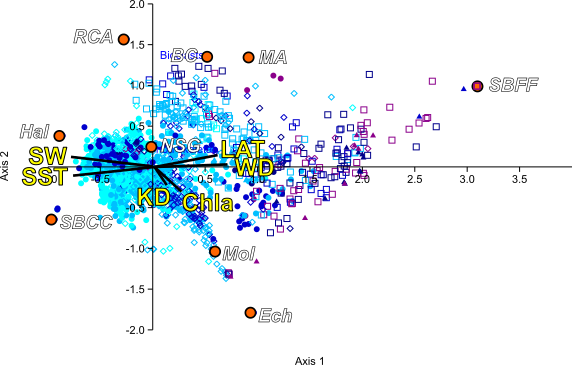


**Figure S3**: Output CCA analysis of the dataset excluding foraminifera. While some changes are present relative to the reference run (Figure 4a,b main text) the main patterns and relation of the other components to one another remains similar.


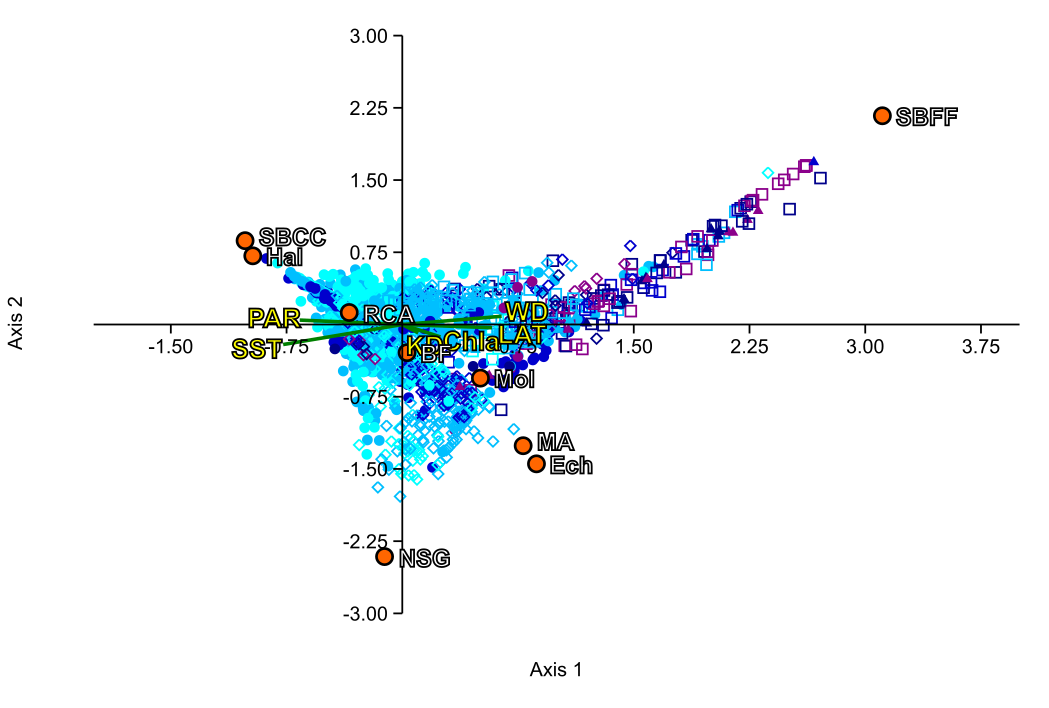


**Figure S4**: Output CCA analysis of the dataset excluding bioclasts. See figure 4 in main text for legend.


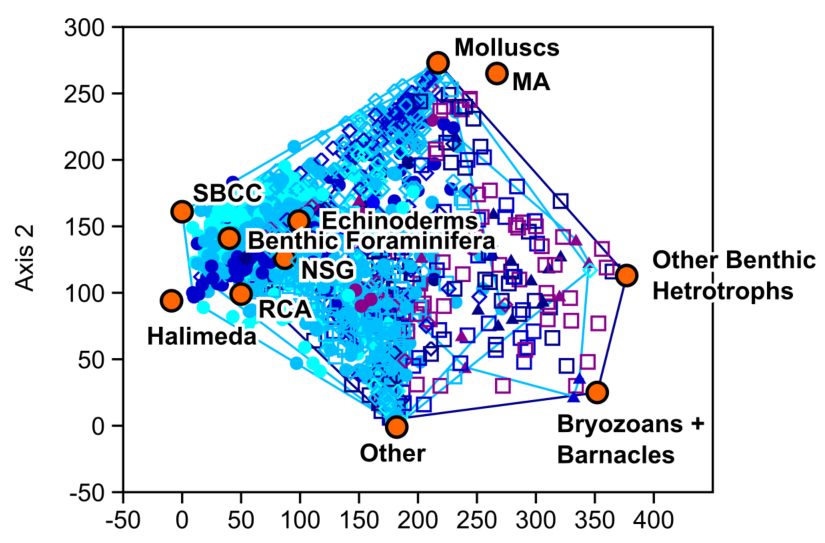


**Figure S5**: Output of DCA analysis with alternative lumping of for sessile benthic filter feeders and other sessile heterotrophs, separating out bryozoans and barnacles. Non identified bioclasts are excluded from this run and only the “other” bioclasts (i.e., the rare types of carbonate producers) are included. See figure 4 in main text for legend.

**Additional references**

1. Martin, T. G. *et al.* Zero tolerance ecology: improving ecological inference by modelling the source of zero observations. *Ecol. Lett.* **8**, 1235–1246 (2005).

2. Hallock, P. & Glenn, E. C. Larger Foraminifera: A Tool for Paleoenvironmental Analysis of Cenozoic Carbonate Depositional Facies. *Palaios* **1**, 55 (1986).

3. Granier, B. The contribution of calcareous green algae to the production of limestones: a review. *Geodiversitas* **34**, 35–60 (2012).

4. Good, S. A., Embury, O., Bulgin, C. E. & Mittaz, J. ESA Sea Surface Temperature Climate Change Initiative (SST_cci): Level 4 Analysis Climate Data Record, version 2.1. *Cent. Environ. Data Anal.* (2019) doi:10.5285/62c0f97b1eac4e0197a674870afe1ee6.

5. Sathyendranath, S. *et al.* Monthly climatology of global ocean colour data products, Version 5.0. *NERC EDS Cent. Environ. Data Anal.* (2021) doi:10.5285/1dbe7a109c0244aaad713e078fd3059a.

6. Hammer, Ø. & Harper, D. A. T. *Paleontological Data Analysis*. *Paleontological Data Analysis* (Blackwell Publishing Ltd., 2007). doi:10.1002/9780470750711.

7. R Core Team. R: A Language and Environment for Statistical Computing. *http://www.r-project.org/* (2020).

8. Sutton, T., Dassau, O. & QGIS Team. QGIS 3.16. at github.com/qgis/QGIS (2021).

9. Pearson, K. LIII. On lines and planes of closest fit to systems of points in space. *London, Edinburgh, Dublin Philos. Mag. J. Sci.* **2**, 559–572 (1901).

10. Shepard, R. N. The analysis of proximities: Multidimensional scaling with an unknown distance function. I. *Psychometrika* **27**, 125–140 (1962).

11. Hill, M. O. *DECORANA - A FORTRAN program for detrended correspondence analysis an reciprocal averaging*. (Section of Ecology and Systematics, Cornell University, 1979).

12. ter Braak, C. J. F. Canonical Correspondence Analysis: A New Eigenvector Technique for Multivariate Direct Gradient Analysis. *Ecology* **67**, 1167–1179 (1986).
